# Loneliness and isolated living status in middle-aged and older adults in Taiwan: exploration on stress-related biomarkers, depressive symptoms, and disability

**DOI:** 10.1186/s12888-022-03824-3

**Published:** 2022-03-12

**Authors:** Tsung-Yu Tsai, Ching-Ju Chiu, Tzu-Yun Wang, Huai-Hsuan Tseng, Kao-Chin Chen, Po-See Chen, Yen-Kuang Yang

**Affiliations:** 1 Department of Psychiatry, National Cheng Kung University Hospital, College of Medicine, National Cheng Kung University, Tainan, Taiwan; 2 Department of Psychiatry, Tainan Hospital, Ministry of Health and Welfare, Tainan, Taiwan; 3 Institute of Gerontology, College of Medicine, National Cheng Kung University, 138 Sheng-Li Road, Tainan, 70428 Taiwan; 4 Institute of Behavioral Medicine, College of Medicine, National Cheng Kung University, Tainan, Taiwan

**Keywords:** Loneliness, Isolated living status, Stress-related biomarkers, Depressive symptoms, Physical disability

## Abstract

**Purpose:**

Loneliness is a subjective feeling by which an individual perceives a lack of closeness in interpersonal relationships. An isolated living status is linked with higher odds of risky health behavior. The conflicting impacts of loneliness and isolated living status on stress-related biomarkers, depressive symptoms, and disability remain unexplained.

**Methods:**

Six hundred twenty-nine participants aged 66.0 (SD=7.3) separated into four groups: “Lonely and Isolated,” “Not Lonely, but Isolated,” “Lonely, but Not Isolated,” and “Neither Lonely, nor Isolated,” were retrieved from the Social Environment and Biomarkers of Aging Study conducted in 2000. Follow-up health indicators in 2006 included three stress-related biomarkers, depressive symptoms, and two physical disability indicators. A hierarchical regression was performed for the analysis.

**Results:**

Firstly, compared to the “Neither Lonely nor Isolated” group, only the “Lonely, but Not Isolated” participants at baseline retained positive associations with the stress-related biomarkers levels 6 years later (urine cortisol level (B=9.25, 95% CI=3.24-15.27), serum Interleukin-6 level (B=2.76, 95% CI=0.72-4.79) and the serum high sensitivity C-reactive protein (hsCRP) level (B=0.40, 95% CI=0.17-0.62)). However, such associations were not observed in the “Lonely and Isolated” participants. Secondly, only “Lonely and Isolated” participants at baseline were positively associated with depressive symptoms 6 years later (B=1.70, 95% CI=0.11-3.30). Finally, the associations between combinations of loneliness and isolated living status and physical disability were eliminated after adjusting the covariables.

**Conclusion:**

Four combinations of loneliness and isolated living status were associated with different impacts on stress-related biomarkers, depressive symptoms, and physical disability. Further dynamic investigations are warranted.

## Background

Living with active social participation is considered a modifiable determinant to reduce the adverse health effects of loneliness and social isolation. However, in Taiwan, 9-20% of older people live alone, and 21% of older adults reported loneliness in 2017 [1]. In addition, according to reports from the World Health Organization, the world’s population over 60 years will increase from 12% in 2015 to 22% in 2050 [2]. Older adults are more likely to have risk factors, such as the death of loved ones and worsening health, that can cause or exacerbate loneliness or social isolation [3]. Therefore, the impacts of loneliness and social isolation on the aging population are becoming increasingly more extensive.

Loneliness is defined as a distressing feeling that occurs due to the discrepancy between desired and available relationships. It is a subjective measure, where an individual perceives a lack of closeness or depth in interpersonal relationships [4]. The prevalence of loneliness has been recognized to range from 7% to 49% in the aging population [5]. As previously reported, up to a 50% higher prevalence of loneliness has been observed in individuals older than 80 [6]. Loneliness is predictive of increased morbidity and mortality [7]. A significant impact of loneliness on physical and mental health has also been found, where more severe levels of loneliness are associated with higher risks of coronary heart disease, increases in depressive symptoms, suicide, cognitive impairment, and functional disability [8]. Personality characteristics, such as neuroticism, have been shown to increase the risk of loneliness and to moderate the risk of depression [9, 10]. In addition, greater loneliness is associated with higher levels of Interleukin-6(IL-6), C-reactive protein (CRP) [11], and lower responsivity of cortisol [12]. Higher levels of Il-6, CRP, and dysregulation of the cortisol response are linked with cardiovascular disease and depression [13]. A person who feels lonely will tend to utilize health services excessively, which will result in an additional financial burden on the medical system [5].

Social isolation is an objective measure of limited social contact between an individual and society, and it is often measured based on social network size, diversity, or frequency of social activity [14]. People suffering from social isolation are at an increased risk of overt diabetes, coronary heart disease, dementia, and increases in the rate of all-cause mortality [15]. Those who experience social isolation will experience an increase in hypothalamic-pituitary-adrenocortical axis (HPA axis) activation and higher levels of inflammatory markers, where such effects are more dependent on the disruption of a social bond between a significant pair than objective isolation per se [16]. In previous studies, researchers reported that about one third of the elderly population lived alone in developed countries from 2000 and 2010 [17]. Living alone in later life may be correlated with a variety of factors, including death of the spouse, divorce, poorer physical or mental health, and personal choice. However, those who begin to live alone after a divorce or the death of a spouse have been shown to have a higher risk of mortality compared with those who live alone for other reasons [18]. In addition, social network structure and function are strongly intertwined with anxiety and depressive symptoms in older adults. For example, one longitudinal mediation analysis showed that social isolation is predictive of higher levels of loneliness and depression among older Americans [19]. The relationship between loneliness and depression may be bi-directional, which often worsens lonely individuals’ health and social activity levels even further [3].

Although loneliness and social isolation have been linked with depression, cardiovascular disease and declined physical function, when researchers simultaneously examine loneliness and social isolation with health, the results are often mixed [20]. People can feel lonely without being isolated and can feel lonely despite living with others. A comparison of subjective individual-level factors and objective environment-related factors showed that loneliness in older adults is higher in the most deprived environments independent of individual-level factors [21]. However, loneliness and social isolation have only been found to be moderately correlated [22]. The potential grouping of people characterized by this viewpoint have not been explored in detail, and measurements of social isolation remain inconsistent [22]. There is a lack of research examining loneliness and an isolated living status (living alone and unmarried), and how these groups might be linked with health.

Assuming that loneliness and an isolated living status may act independently and lead to different health trajectories through their effects on health-risk behaviors, this study is aimed toward exploring the inconsistent findings concerning the role of risk of loneliness and isolated living status on stress-related biomarkers, depressive symptoms, and disability. The participants were separated into four groups [22]: lonely and isolated; lonely but not isolated; not lonely, but isolated; not isolated and not lonely to elaborate on the different impacts of loneliness and isolated living status on health outcomes.

## Methods

### Participants

**Fig. 1:**
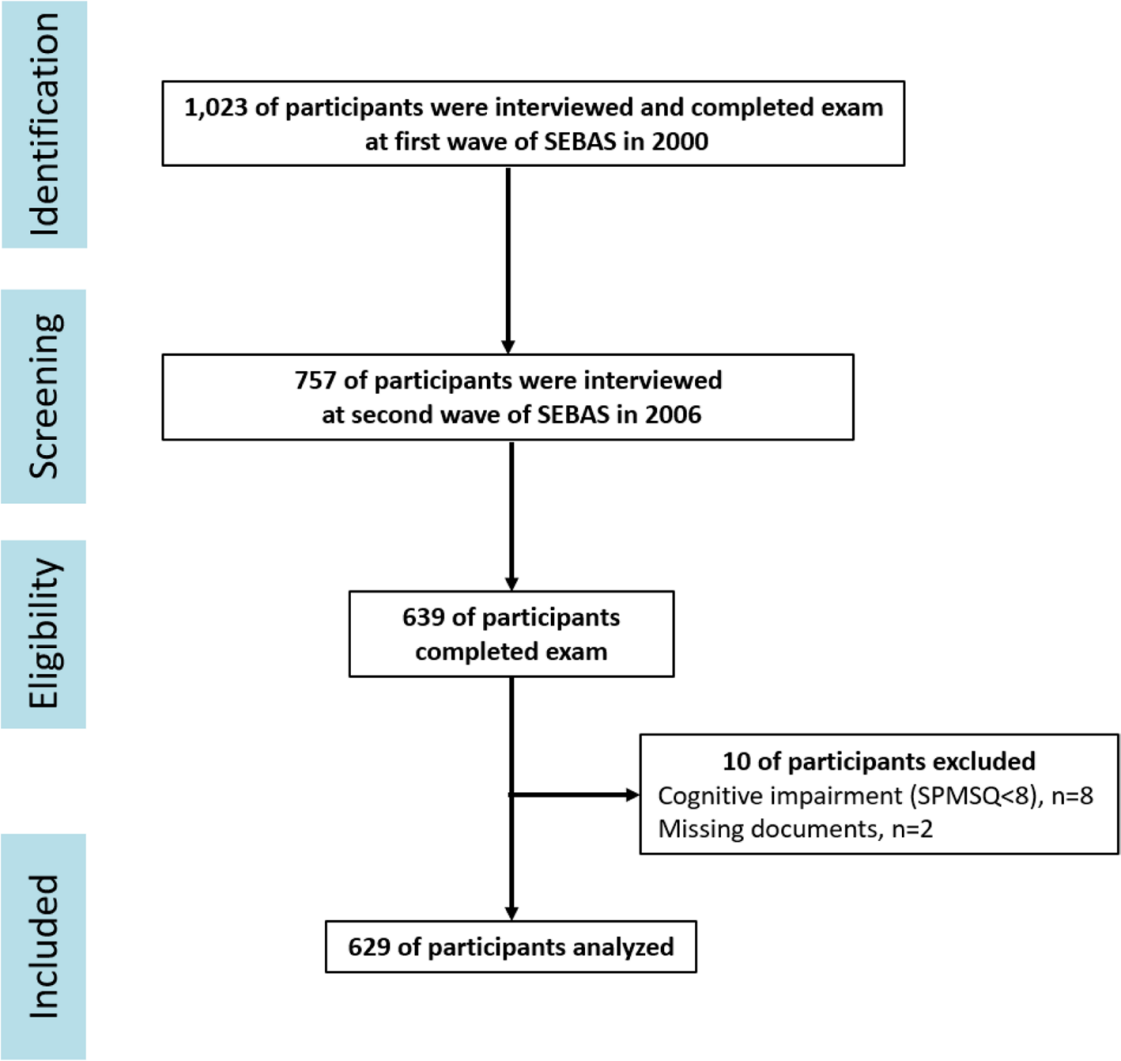
Flow chart of sample selection, participation, and attrition for SEBAS 2000 and 2006

Six hundred twenty-nine participants were enrolled from the Social Environment and Biomarkers of Aging Study (SEBAS 2000 and 2006) in this study. The SEBAS is an extension of the Taiwan Longitudinal Study on Aging (TLSA), which began in 1989 and has undergone repeated follow-ups every 3-4 years with a nationally representative sample of adults aged 60 and older. Younger refresher cohorts of the TLSA were added in 1996 and 2003, and as of 1996, participants in the TLSA were representative of older adults aged 50 and older in Taiwan. The first wave of the SEBAS, based on a sub-sample of participants from the 1999 TLSA, was conducted in 2000. A total of 1,713 participants aged 54 and over in 27 townships were selected from the TLSA 1999. There were 1,023 participants who had been interviewed and completed a hospital-based health examination in the first wave of the SEBAS in 2000. The second wave of SEBAS was conducted in 2006 using a protocol similar to that for SEBAS 2000. In both SEBAS waves, health status, health behavior, exposure to stressors, and social relationships were collected. With the exception of participants who passed away or were lost to follow-up, there were 757 participants who had been interviewed for the SEBAS 2006, and 639 of them had completed the health and hospital-based examination assessment [23]. To ensure the reliability of the self-report questionnaire used in the present study, we had to exclude 8 participants who might have had cognitive function impairments based on Short Portable Mental State Questionnaire (SPMSQ) scores≦7, as well as 2 participants with missing documents. Therefore, there was a total of 629 participants aged 54 and over at baseline and with interview and biomarker data in both 2000 and 2006 analyzed in this study (Fig. 1).

### Measures

#### Explanation of the variables, including loneliness and isolated living status

Loneliness was assessed in 2000 and 2006 by asking the participants “In the past week, have you experienced the following situations or feelings of loneliness (Felt isolated, with no companions)?” The four answer options were “never,” “rarely (1 day),” “sometimes (2-3 days),” and “often (>4days).” Responses were recorded as a 4-point Likert-type response, ranging from “never” to “often.” Participants who answered “never” were categorized as the non-lonely group. Those who answered “rarely,” “sometimes (2-3 days),” or “often” were grouped together as the “lonely” group.

Multidimensional measures of the Isolated Living Status Index indicate that different aspects of social interaction may have a relationship with depressive symptoms and stress-related biomarkers [24]. Indicators of social isolation in a previous study included living alone, being unmarried, low participation in social activities, and infrequent contact with network members [25]. In the present study, two items (married and living alone) were used to develop an index of isolated living status in 2000. One point was assigned to participants who were not married/separated/divorced/widowed. One point was assigned to participants who were living alone. These two items were added together to obtain an overall isolated living status index ranging from 0-2. All participants who responded ‘married, and not living alone’ were classified as “non-isolated living status” (isolated living status index=0). All participants who had Isolated Living Status Index scores ≧1 were classified as experiencing isolation.

Combining loneliness with living status, four categories of loneliness and living status were identified: 1. Lonely and Isolated, 2. Not Lonely, but Isolated, 3. Lonely, but Not Isolated, 4. Neither Lonely, nor Isolated. To compare within-group differences, Neither Lonely, nor Isolated was used as a reference category.

#### The three domains of the outcome variables: stress-related biomarkers (Cortisol, IL-6, and hsCRP), depressive symptoms, and disability (Mobility; IADL)

Firstly, in the SEBAS 2000 and 2006, overnight and 12-hour urine specimens (7pm to 7am) was collected to minimize person-to-person variations and diurnal variations, which provided a more accurate measurement of baseline levels of stress-related biomarkers. The participants provided the urine specimen, and a phlebotomist drew a blood sample. Data from duplicate samples indicated intra-lab correlations of 0.8 or higher and inter-lab correlations of 0.6 or higher.

Urine cortisol, measured by using high-performance liquid chromatography (HPLC) in both the 2000 and 2006 SEBAS, were used to assess HPA activity. Depression was linked with higher degrees of dysregulation of HPA activity and higher basal cortisol levels. By contrast, higher levels of cortisol were noted when older adults felt acutely lonely, where their HPA activity would be blunted in the chronic phase [26, 27]. IL-6 was measured using enzyme-linked immunoassays (EIA; Endogen, Pierce Biotechnology) in the SEBAS 2000, as well as enzyme-linked immunosorbent assays (ELISA; R&D Systems). Measurements using EIAs and ELISAs are virtually the same despite the reagent kit having different manufacturers. An ELISA provides more precise sensitivity than an EIA. High-sensitivity C-reactive protein (hsCRP) was measured using an immunoturbidimetry (Roche Cobas Integra 800) method in the SEBAS 2006.

Secondly, the ten-item Center for Epidemiologic Studies Depression Scale (CES-D) is a 4-point scale ranging from 0 to 3 [28]. Participants indicated if, during the past 2 weeks, they had experienced a given symptom or not. A total CES-D score was added across the 9 items in the present study: Not interested in eating/poor appetite, doing anything was exhausting, slept poorly, was in a terrible mood, people were not nice to me, felt anxiety, no will/energy to do things, felt joyful, felt life was going well. One item was used to indicate loneliness that was not included as a depressive symptom. The Cronbach’s alpha of the 9-item CES-D was 0.80 in 2000 and 0.81 in 2006. A higher total CES-D score indicated a higher level of depressive symptoms.

Lastly, physical disabilities were measured in 2000 and 2006 with two indicators, including a modified 9-item strength and mobility activities scale [29], and the 6-item Instrumental Activities of Daily Living (IADL) scale [30]. The mobility scale is a 4-point scale ranging from 0 to 3 (0=no difficulty, 3=unable to do it). The participants responded to the following questions: “If no one helps you, and you do not use aids, would you have difficulty doing the following activities by yourself?” A total mobility score was added across the 9 items: Stand for 15 minutes; stand for 2 hours; squat; raise both hands over the head; grasp objects with fingers, lift/carry 11-12 kilograms; run 20-30 minutes; walk 200-300 meters, and walk up 2-3 flights of stairs. The IADL scale is a 4-point scale ranging from 0 to 3 (0=no difficulty, 3=unable to do it). The Cronbach’s alpha of the mobility scale was 0.86 in 2000 and 0.88 in 2006. Participants responded to the following question: “Based on your health and physical condition, do you have difficulty doing the following activities by yourself?” A total IADL score was added across the 6 items: buying personal items, managing money, riding a bus/train alone, doing physical work at home, doing light housework, and making phone calls. Higher IADL and mobility scores indicated greater functional limitations. A score of zero indicated no disabilities. The Cronbach’s alpha of IADL scale was 0.76 in 2000 and 0.89 in 2006.

### Confounding factors

Participants were asked the following: “Do you still have this illness now?” One point was assigned if the participants answered “Yes, I still have this illness now.” Total 9 items of comorbidity including high blood pressure, diabetes, heart disease, stroke, cancer/malignant tumor, lower respiratory disease, arthritis/rheumatism, liver/gall bladder disease, and kidney disease were collected.

Cognitive function was evaluated using the Short Portable Mental State Questionnaire (SPMSQ, Cronbach’s alpha =0.77) in 2000 [31]. Participants were asked, “Tell me your address or where this is?,” “What is today’s date (year)?,” “What is today’s date (month)?,” “What is today’s date (day)?,” “What day of the week is it?,” “How old are you this year?,” “What is your mother’s surname?,” “Who is the President?,” “Who were the previous presidents of Taiwan?” along with the serial 3s subtraction task. Higher scores indicated higher cognitive ability. If the participants had more than three errors, they were suspected of having cognitive impairment. Participants who had SPMSQ scores≦7 were excluded in the SEBAS 2000 because of potential cognitive impairment.

Personal stress was assessed by using the 10-item Perceived Stress Scale (PSS, Cronbach’s alpha=0.81) in 2006 [32]. The PSS is a 5-point scale ranging from 0 to 4 (0=never, 4=always). Participants were asked, during the last month, “How often upset by unexpected events?,” “How often felt unable to control important things?,” “How often felt nervous or stress?,” “How often could you not cope with all had to do?,” “How often have been angered by things outside your control?,” “How often felt difficulties so bad they could not be overcome?,” “How often felt confident about handling personal problems?,” “How often felt thing going your way?,” “How often been able to control irritations in your life?,” and “How often felt you were on the top of things?” The last 4 question scores have been reversed as below: 0=4, 1=3, 2=2, 3=1, 4=0. Higher 10-item PSS scores indicated greater perceived stress.

Socio-demographic variables included age, sex, education (No schooling, 1-6 years, 7-9 years, 9-12 years,≧12 years).

### Statistical analyses

Analyses were carried out with SAS 9.4. statistical software and included descriptive statistics and a hierarchical linear regression. Descriptive analyses were performed for all variables at baseline and 6 years later. We used Student’s *t-*tests and Pearson’s correlations to assess the associations between the demographic data and the outcome variables (cortisol, IL-6, and hsCRP, CES-D, mobility, and IADL). Furthermore, paired t-tests and McNemar’s test were used to assess associations between the two waves of the SEBAS. All tests were evaluated at a 0.05 level of statistical significance.

We applied hierarchical regression models to investigate the association between different combinations of baseline loneliness and isolated living status and three outcome variable domains 6 years later: stress-related biomarkers (cortisol, IL-6, and hsCRP), depressive symptoms, and physical disability (mobility/IADL disability). For each outcome variable, three models were estimated. In Model 1, we regressed the combinations of baseline loneliness and isolated living status on the outcome variables 6 years later. In Model 2, we then repeated the analyses, adjusting for the baseline outcome variables. In Model 3, we repeated the analyses adjusting for confounding variables such as age, sex, educational attainment, baseline depressive symptoms, PSS score 6 years later, and comorbidity 6 years later. In terms of statistical power, only the comorbidity item accounting for more than 5% of participants was treated as an independent variable in Model 3.

The results are presented as regression coefficients (B) with a 95% confidence interval.

## Results

The characteristics of the sample are summarized in Table 1. The mean age of this study population was 66.0 years (SD=7.3) at baseline, and 58.8% of the sample was male, 77.9% of the participants were married; 5.7% lived alone, and 14.5% were experiencing loneliness. The outcome variables at baseline and 6 years later were as follows: urine cortisol level (baseline: mean = 19.7±18.3ug/L, 6 years later: mean = 12.1±17.2, *p* < 0.001), serum IL-6 level (baseline: mean = 2.9±3.3 pg/mL, 6 years later: mean = 4.0±6.0, *p* < 0.001), serum hsCRP (baseline:-, 6 years later: mean = 0.3±0.6mg/dl), CES-D score (baseline: mean = 4.7±4.8, 6 years later: mean = 4.9±5.2, *p* = 0.39), mobility disability (baseline: mean = 2.7±4.2, 6 years later: mean = 5.1±6.2, *p* = <0.001), and IADL disability (baseline: mean=0.6±1.8, 6 years later: mean = 2.0±3.6, *p* = <0.001). To examine for potential bias and collinearity, a Pearson’s correlation of all variables, including age and education at baseline, and PSS, CES-D, mobility, IADL index scores, cortisol, IL-6, and hsCRP 6 year later were performed, as summarized in Table 2. The baseline descriptive statistics for the four groups, Lonely and Isolated, Not Lonely, but Isolated, Lonely, but Not Isolated, and Neither Lonely, nor Isolated, are presented in Table 3. Also, Table 3 shows the group differences, adjusted using the Bonferroni correction for pairwise comparisons of the four groups at baseline, on age (*F* = 12.2, *p* < 0.001), sex (χ^2^ = 30.5, *p* < 0.001), comorbidity (*F* = 4.3, *p* < 0.001), urine cortisol level (*F* = 4.2, *p* = 0.001), serum IL-6 level (*F* = 0.8, *p* = 0.51), CES-D score (*F* = 51.4, *p* <0.001), mobility disability (*F* = 20.3, *p* < 0.001), and IADL disability (*F* = 10.8, *p* < 0.001). The group differences, adjusted using the Bonferroni correction for pairwise comparisons of the four groups 6 years later, were as follows: PSS score (*F*=9.1, *p*<0.001), urine cortisol level (*F* = 4.5, *p* = 0.004), serum IL-6 level (*F* = 3.5, *p* = 0.02), serum hsCRP level (*F* = 7.7, *p* < 0.001), CES-D score (*F* = 11.0, *p* < 0.001), mobility disability (*F* = 9.6, *p*<0.001), and IADL disability (*F* = 7.0, *p* < 0.001)

**Table 1:** Participant Characteristics at Baseline and Follow-up (SEBAS 2000 and SEBAS 2006)

Variable	SEBAS2000	SEBAS2006	Paired *t* or χ^2^	P
Age (years, mean ± SD)	66.0±7.3	72.0±7.2	-203.27	<0.001
Sex (Male/Female)	370/259	370/259	-	-
Marital status (n/%)			34.3	<0.001
Unmarried	139(22.1)	180(28.6)		
Married	490(77.9)	449(71.4)		
Living alone (n/%)			10.8	0.001
Yes	36(5.7)	57(9.1)		
No	593(94.3)	572(90.9)		
Loneliness			0.13	0.72
Yes	91 (14.4)	87 (13.8)		
No	538 (85.6)	542 (86.2)		
Education (n/%)			-	-
No schooling	162 (25.8)	162 (25.8)		
Elementary (<6 yrs) Junior High (7-9 yrs) Senior High (9-12 yrs) College (>12 yrs)	278 (44.2) 68 (10.8) 69 (11.0) 52 (8.2)	278 (44.2) 68 (10.8) 69 (11.0) 52 (8.2)		
Health status (n/%) High blood pressure			56.9	<0.001
Yes	174 (27.7)	259 (41.2)		
No	455 (72.3)	370 (58.8)		
Diabetes			44.6	<0.001
Yes	76 (12.1)	126 (20.0)		
No	553 (87.9)	503 (80.0)		
Heart disease			26.0	<0.001
Yes	85 (13.5)	142 (22.6)		
No	544 (86.4)	487 (77.4)		
Stroke			24.1	<0.001
Yes	8 (1.3)	34 (5.4)		
No	621 (98.7)	595 (94.6)		
Cancer/malignant tumor			2.6	0.10
Yes	4 (0.6)	10 (1.6)		
No	625 (99.4)	619 (98.4)		
Lower respiratory disease			0.06	0.80
Yes	48 (7.6)	50 (8.0)		
No	581 (92.4)	579 (92.0)		
Arthritis/rheumatism			3.6	0.06
Yes	97 (15.4)	118 (18.8)		
No	532 (84.6)	511 (81.2)		
Liver/gall bladder disease			6.5	0.01
Yes	23 (3.7)	41 (6.5)		
No	606 (96.3)	588 (93.5)		
Kidney disease			3.2	0.07
Yes	27 (4.3)	40 (6.4)		
No	602 (95.7)	589 (93.6)		
Perceived Stress Scale	-	9.1±6.4	-	-
Stress-related biomarkers Cortisol levels	19.7±18.3	12.1±17.2	7.64	<0.001
IL-6 levels	2.9±3.3	4.0±6.0	-4.06	<0.001
hsCRP	-	0.3±0.6	-	-
Depression (CES-D)# Disability	4.7±4.8	4.9±5.2	-0.87	0.39
Motility	2.7±4.2	5.1±6.2	-11.8	<0.001
IADLs	0.6±1.8	2.0±3.6	-10.8	<0.001

Note. Numbers are Mean±SD or N (%). *CES-D* The Center for Epidemiologic Studies Depression Scale; *IADLs* Instrumental Activities of Daily Living; *IL-6* Interleukin-6; *hsCRP* High sensitivity C-reactive protein. #9-item CES-D score (excluding lonely). *p < 0.05; **p < 0.01; *** *p* < 0.001

**Table 2:** Correlation Matrix for the Continuous Variables in the Study

	1	2	3	4	5	6	7	8	9
**1. Age**	-	-0.06	-0.05	0.20***	0.43***	0.37***	0.03	0.10	0.03
**2. Education**	-0.12	-	<0.01	-0.13*	-0.17**	-0.11*	-0.07	-0.02	-0.09
**3. PSS**	-0.03	-0.001	-	0.30***	0.16**	0.09	0.07	0.05	0.09
**4. CES-D**^**#**^	0.21***	-0.17**	0.39***	-	0.43***	0.37***	<0.01	0.10	0.02
**5. Mobility**	0.48***	-0.22***	0.15*	0.45***	-	0.76***	<-0.01	0.16**	0.08
**6. IADLs**	0.48***	-0.21***	0.18**	0.40***	0.83***	-	-0.06	0.18**	0.05
**7. Cortisol**	-0.04	-0.07	0.20**	0.12	-0.01	0.08	-	0.07	0.26***
**8. IL-6**	0.18**	-0.17*	0.04	0.07	0.02	0.06	-0.03	-	0.54***
**9. hsCRP**	-0.001	-0.07	0.10	0.16*	0.14*	0.20**	-0.01	0.10	-

Note. The upper diagonal was based on men (*N*=370); the lower diagonal was based on women (*N*=259); *PSS* The Perceived Stress Scale; *CES-D* The Center for Epidemiologic Studies Depression Scale; *IADLs* Instrumental Activities of Daily Living; *IL-6* Interleukin-6; *hsCRP* High sensitivity C-reactive protein. #9-item CES-D score (excluding lonely); *p < 0.05; **p < 0.01; *** *p* < 0.001

**Table 3:** Sociodemographic and Health-related Characteristics of Participants in the Four Loneliness and Isolated Living Status Groups

	^a^Neither Lonely, nor Isolated (*N*=438)	^b^Not Lonely, but Isolated (*N*=100)	^c^Lonely, but Not Isolated (*N*=49)	^d^Lonely and Isolated (*N*=42)	F or χ^2^	Pairwise comparisons among means
Age	65.0±7.2	69.2±7.0	66.2±7.1	69.1±6.8	12.2^***^	a<b, a<d
Sex					30.5^***^	
Male	287(65.5)	39(39)	27(55.1)	17(40.5)		
Female	151(34.5)	61(61)	22(44.9)	25(59.5)		
Education					20.8	
No schooling	97(22.2)	29(29)	20(40.8)	16(38.1)		
Elementary (<6 years)	195(44.5)	46(46)	17(34.7)	20(47.6)		
Junior High (7-9 years)	53(12.1)	10(10)	2(4.1)	3(7.1)		
Senior High (9-12 years)	50(11.4)	11(11)	6(12.2)	2(4.8)		
College (>12 years)	43(9.8)	4(4)	4(8.2)	1(2.4)		
Comorbidity^+^	0.8±0.9	0.9±1.0	1.1±1.1	1.2±1.2	4.3^***^	a<d
Loneliness^2006^						
Yes	43(9.8)	17(17.0)	10(20.4)	17(40.5)		
No	395(90.2)	83(83.0)	39(79.6)	25(59.5)		
Isolated living status^2006^						
Yes	39(8.9)	99(99.0)	10(20.4)	41(97.6)		
No	399(91.1)	1(1.0)	39(79.6)	1(2.4)		
Perceived Stress Scale	8.6±5.9	8.6±6.9	13.4±7.1	11.5±7.7	9.1^***^	b<c, d<c
Stress-related Biomarkers						
Cortisol levels^2000^	21.2±20.4	16.6±13.3	18.3±10.6	12.7±7.9	4.2^**^	a>d
Cortisol levels^2006^	11.9±15.7	9.2±13.2	21.1±26.7	10.1±23.6	4.2^**^	a<c, b<c
IL-6 levels^2000^	2.9±3.3	2.9±2.4	2.7±2.7	3.7±4.7	0.8	
IL-6 levels^2006^	3.7±5.3	3.9±4.3	6.6±11.2	5.1±7.4	3.5^*^	a<c
hsCRP^2000^	-	-	-	-	-	-
hsCRP^2006^	0.3±0.5	0.3±0.5	0.8±1.6	0.3±0.3	7.67^***^	a<c, b<c, d<c
Depression^#^						
CES-D^2000^	3.8±4.0	4.3±3.8	10.5±6.1	9.2±6.0	51.4^***^	a<c, a<d, b<c, b<d
CES-D^2006^	4.3±4.7	5.4±5.5	6.6±5.4	8.5±7.1	11.0^***^	a<c<d, b<d
Disability						
Mobility^2000^	2.0±3.4	3.7±4.3	5.3±6.0	5.3±5.5	20.3^***^	a<b, a<c<d
Mobility^2006^	4.3±5.6	6.7±6.7	6.3±7.0	8.3±7.3	9.6^***^	a<b, a<d
IADLs^2000^	0.4±1.5	1.0±2.1	1.5±2.6	1.4±2.1	10.8^***^	a<b, a<c, a<d
IADLs^2006^	1.6±3.2	2.9±4.4	2.5±4.4	3.5±4.3	7.0^**^	a<b, a<d

Note. Numbers are Mean±SD or N (%). *CES-D* The Center for Epidemiologic Studies Depression Scale; *IADLs* Instrumental Activities of Daily Living; *IL-6* Interleukin-6; *hsCRP* High sensitivity C-reactive protein. +total 9-item comorbidity score. #9-item CES-D score (excluding lonely). *p < 0.05; **p < 0.01; *** *p* < 0.001

### Effect of baseline loneliness and isolated living status on stress-related biomarkers 6 years later

First of all, as shown in Table 4, Model 1 on the levels of urine cortisol and serum IL-6 indicated that only the Lonely, but Not Isolated group was positively associated with greater levels of urine cortisol (B = 9.11, 95% CI = 3.47-14.75) and serum IL-6 (B = 2.96, 95% CI = 1.02-4.91) 6 years later. Model 2 further added the baseline levels, and the positive association remained (Cortisol levels: B = 9.34, 95% CI=3.97-14.95; IL-6 levels: B = 3.20, 95% CI = 1.31-5.09). After adjusting for age, sex, education, depressive symptoms at baseline, and comorbidity (high blood pressure, diabetes, heart disease, lower respiratory disease, and arthritis/rheumatism) 6 years later in Model 3, the Lonely, but Not Isolated group retained a consistently positive association with urine cortisol levels (B = 9.25, 95% CI = 3.24-15.27) and serum IL-6 levels 6 years later (B = 2.76, 95% CI = 0.72-4.79).

**Table 4:** Four-Combination Effects of Baseline Loneliness and Isolated Living status on Stress-related Biomarkers, Depressive Symptoms, and Disability 6 Years Later

	Cortisol			IL-6			hsCRP		
	B(95%CI)	R^2^	p	B(95%CI)	R^2^	p	B(95%CI)	R^2^	p
Model 1: Unconditional		*0.03*	*0.004*		*0.02*	*0.02*		*0.04*	*<0.001*
Lonely and Isolated	-1.79 (-7.79 to 4.21)			1.47 (-0.52 to 3.46)			-0.003 (-0.21 to 0.21)		
Not Lonely, but Isolated	-2.70 (-6.85 to 1.45)			0.24 (-1.19 to 1.67)			0.04 (-0.12 to 0.19)		
Lonely, but Not Isolated	**9.11 (3.47 to 14.75)**			**2.96 (1.02 to 4.91)**			**0.50 (0.30 to 0.71)**		
Model 2: Baseline adjusted		0.04	<0.001		0.11	<0.001		-	-
Lonely and Isolated	-0.60 (-6.62 to 5.42)			0.99 (-0.92 to 2.91)			-		
Not Lonely, but Isolated	-2.01 (-6.17 to 2.14)			0.25 (-1.14 to 1.64)			-		
Lonely, but Not Isolated	**9.34 (3.73 to 14.95)**			**3.20 (1.31 to 5.09)**			-		
Model 3: Full adjusted		0.08	<0.001		0.13	<0.001		0.07	<0.001
Lonely and Isolated	0.41 (-6.06 to 6.89)			0.42 (-1.68 to 1.63)			-0.09 (-0.32 to 0.14)		
Not Lonely, but Isolated	-1.60 (-5.94 to 2.74)			-0.06 (-1.53 to 1.41)			0.05 (-0.11 to 0.21)		
Lonely, but Not Isolated	**9.25 (3.24 to 15.27)**			**2.76 (0.72 to 4.79)**			**0.40 (0.17 to 0.62)**		
	#CES-D score			Mobility			IADL		
	B(95%CI)	R^2^	p	B(95%CI)	R^2^	p	B(95%CI)	R^2^	p
Model 1: Unconditional		*0.05*	*<0.001*		*0.04*	*<0.001*		*0.03*	*<0.001*
Lonely and Isolated	**4.15 (2.54 to 5.77)**			**4.07 (2.15 to 5.98)**			**1.92 (0.78 to 3.06)**		
Not Lonely, but Isolated	**1.14 (0.03 to 2.25)**			**2.38 (1.07 to 3.70)**			**1.35 (0.57 to 2.13)**		
Lonely, but Not Isolated	**2.31 (0.80 to 3.82)**			**2.02 (0.23 to 3.81)**			0.95 (-0.11 to 2.02)		
Model 2: Baseline adjusted		0.13	<0.001		0.36	<0.001		0.29	<0.001
Lonely and Isolated	**2.34 (0.72 to 3.97)**			1.18 (-0.43 to 2.79)			0.90 (-0.09 to 1.89)		
Not Lonely, but Isolated	0.97 (-0.10 to 2.03)			0.86 (-0.24 to 1.95)			**0.74 (0.06 to 1.41)**		
Lonely, but Not Isolated	0.07 (-1.49 to 1.64)			-0.90 (-2.41 to 0.60)			-0.25 (-1.18 to 0.68)		
Model 3: Full adjusted		0.22	<0.001		0.41	<0.001		0.35	<0.001
Lonely and Isolated	**1.70 (0.11 to 3.30)**			0.19 (-1.41 to 1.79)			0.29 (-0.65 to 1.22)		
Not Lonely, but Isolated	0.48 (-0.62 to 1.58)			-0.35 (-1.45 to 0.75)			0.10 (-0.54 to 0.75)		
Lonely, but Not Isolated	-0.61 (-2.15 to 0.92)			-1.15 (-2.70 to 0.39)			-0.29 (-1.19 to 0.61)		

Note. The “Neither Lonely, nor Isolated” group was the reference group. 2. *CES-D* The Center for Epidemiologic Studies Depression Scale; *IADL* Instrumental Activities of Daily Living; IL-6 Interleukin-6; *hsCRP* High sensitivity C-reactive protein. #9-item CES-D score (excluding lonely). 3. Bold numbers indicate significance (*p* < 0.05)

Lastly, because of limited data on serum hsCRP levels in the SEBAS 2000, we performed two steps in the regression model to assess the associations between combinations of loneliness and isolated living status at baseline and the serum hsCRP levels 6 years later. As shown in Table 4, Model 1 on the serum hsCRP levels indicated that only the Lonely, but Not Isolated (B = 0.50, 95% CI = 0.30-0.71) group was positively associated with greater serum hsCRP levels 6 years later. After adjusting for other controls, including age, sex, education, depressive symptoms at baseline, PSS 6 years later, and comorbidities 6 years later in the second step, the Lonely, but Not Isolated group at baseline remained consistently positively associated with serum hsCRP levels 6 years later (B = 0.40, 95% CI = 0.17-0.62).

### Effect of baseline loneliness and isolated living status on depressive symptoms 6 years later

As shown in Table 4, Model 1 on depressive symptoms showed that both loneliness and isolated living status were associated with a higher CES-D score (Table 4). Model 2 further added the CES-D scale at baseline, and we observed that only when loneliness and isolated living status occurred together (the Lonely and Isolated group) were the CES-D scores higher (B = 2.34, 95% CI = 0.72-3.97). The positive association between loneliness and isolated living status and the CES-D scores remained robust after further adjustment for age, sex, education, PSS 6 years later, and comorbidities 6 years later (B = 1.70, 95% CI = 0.11-3.30).

### Effect of baseline loneliness and isolated living status on physical disability 6 years later

As shown in Table 4, Model 1 on mobility disability indicated that the Lonely and Isolated (B=4.07, 95% CI=2.15-5.98), Not Lonely, But Isolated (B=2.38, 95% CI=1.07-3.70), and Lonely, Not Isolated (B = 2.02, 95% CI = 0.23-3.81) groups had a positive association with greater levels of mobility disability 6 years later. Model 2 further added mobility disability at the baseline, and the previous associations were eliminated. Furthermore, after adjusting for age, sex, education, depressive symptoms at baseline, PSS 6 years later, and comorbidities 6 years later, there were no significant differences found among all combinations of baseline loneliness and isolated living status and mobility disabilities 6 years later.

Furthermore, as shown in Table 4, Model 1 on IADL disability indicated that the Lonely and Isolated (B = 1.92, 95% CI = 0.78-3.06) and Not Lonely, But Isolated (B = 1.35, 95% CI = 0.57-2.13) groups were associated with a higher IADL disability score 6 years later. Subsequently, Model 2 added the baseline IADL score, and only the Not Lonely, But Isolated group remained positively associated with the IADL disability score 6 years later (B = 0.74, 95% CI = 0.06-1.41). However, this association vanished after further adjusting for age, sex, education, depressive symptoms at baseline, PSS 6 years later, and comorbidities 6 years later.

## Discussion

In this study, compared to the Neither Lonely, Nor Isolated group, only people who lived with others and/or got married still experienced loneliness and were prone to having higher levels of cortisol, IL-6, and hsCRP. Secondly, people experiencing both loneliness and an isolated living status were found to have more depressive symptoms, independent of their age, sex, education, number of comorbidities, stress, and baseline depressive symptoms. However, neither loneliness nor isolated living status was found to be associated with development of depressive symptoms. In terms of physical disability, we found that higher levels of loneliness and isolated living status were associated with higher levels of mobility and IADL disabilities. However, after adjusting for the confounding variables, neither loneliness nor isolated living status was found to be related to the levels of mobility disability or IADL disability.

### Synergic effects of loneliness and isolated living status on stress-related biomarkers, depressive symptoms, and physical disability

Loneliness and isolated living status have been linked with the Hypothalamic-Pituitary-Adrenal axis and systemic inflammation. In our study, although we found a combination of loneliness and isolated living status to be associated with higher levels of depressive symptoms, this association between loneliness and isolated living status at baseline and the stress-related biomarkers was not observed 6 years later. The theory of allostasis and the allostatic load in social dynamics, stress, and physiological responses may explain this phenomenon [33]. Participants who are socially isolated exhibit dysregulated patterns of mood and physiology. The theory of allostatic load means that chronic, sustained stress creates wear and tear on regulatory systems [34]. Our findings suggest that lonely and isolated participants may have failed to regulate their emotions within groups due to an increased allostatic load, which led to depression and attenuated inflammatory responses.

Furthermore, we reaffirmed the hypothesis that people who are both lonely and isolated rather than lonely or isolated individually are at higher risk for the development of depression. Some investigators have suggested that gender differences in individuals living alone may be a predictor of the gender differences found in depression. Older women are more likely than older men to be unmarried (widowed/divorced/separated) and to live alone than older men. However, older men who live alone have more depressive symptoms than older women who live alone [35]. Men also reported more loneliness than women [36]. In our study, older men who were lonely and socially isolated tended to have higher levels of depressive symptoms (B = 3.15, *p* = 0.01), but this association was not significant in women (B = -0.15, *p* = 0.90) after adjusting for the confounding variables.

The relationships between loneliness, isolated living status, and physical disability in older individuals have remained unclear and inconsistent. Some studies have reported that having a large number of social relationships is associated with fewer physical disabilities [37], but some studies have reported limited or no significant associations [38]. In one cross-sectional study examining the combined effect of marital status and living arrangement, married older adults living with children had better IADL scores than those who were unmarried and living with children [39]. In addition, feelings of loneliness may exacerbate existing vulnerabilities in health that lead to disabilities, either though poor health behavior or through an inflammatory or cardiovascular pathway. A later prospective study showed both social isolation and loneliness to be associated with a decrease in gait speed [40]. In our study, we found that loneliness and isolated living status at baseline were positively correlated with mobility disabilities and IADL disabilities 6 years later. However, these associations were not significant after adjusting for the baseline conditions. One consideration is that socio-economic status may act as a buffer against the effects of social relationships on functional disabilities. Greater social resources were associated with better self-rated health as well as a composite measure of physical function. Even with these findings, the mechanisms remain unclear, and further investigation may be needed.

### Effects of loneliness without an isolated living status on stress-related biomarkers, depressive symptoms, and physical disability

Loneliness affects people at any stage of life. Some participants tend to widen their social network to achieve a desirable level of social interaction, but some of them do not [41]. In our study, we found that the Lonely, but Not Isolated participants at baseline were positively associated with higher levels of depressive symptoms and mobility disabilities 6 years later. However, this association was not observed after adjusting for the baseline conditions. Interestingly, we found a consistent positive association between loneliness and the stress-related biomarker levels (Table 4). A lonely participant with unsatisfactory levels of social support had a high likelihood of psychological distress and an inflammatory response [42]. After a sex stratification analysis, older women who felt lonely but were not socially isolated had higher levels of Cortisol, IL-6, and hsCRP, but such associations were not observed in older men. Loneliness was associated positively with demographic and environmental factors such as physical illness, a small social network, and a lack of a spousal confidant. On the contrary, the same objective social relationships (e.g., spouse) and higher levels of education were found to be protective factors for health. Loneliness and isolated living status were independently associated with lower levels of self-rated physical health. The association between loneliness and isolated living status was mediated by the perceived quality of social relationships. For example, even when these individuals had a spouse, the marital relationship could be tense, which can have negative consequences for individuals [43]. In contrast, active social participation can lead to an increase in physical exercise, alleviated loneliness, and lower levels of physical disability. Active participation in social activities plays an important role in maintaining mental and physical well-being [41].

### Effects of isolated living status without loneliness on stress-related biomarkers, depressive symptoms, and physical disability

Some gaps between loneliness and isolated living status were found for the Only Isolated group just as was the case with the Only Lonely group. A previous longitudinal mediation analysis suggested that social disconnectedness (e.g., unmarried with infrequent social interaction) predicts higher levels of subsequent loneliness, which in turn predict higher levels of depressive symptoms in a general older adult population [19]. A comparison of loneliness and social isolation indicated that loneliness has a stronger association with depressive symptoms than social isolation [44, 45]. In this study, we found that people experiencing isolated living status without loneliness at baseline were not associated with depressive symptoms 6 years later after adjusting for baseline depression. Older adults who are living alone and unmarried may be able to optimize social relationships or perceived deficits in support [46]. Also, if participants tended to prefer being alone to being with others, this desire for solitude may actually reduce stress levels and enhance mental balance [33]. This might imply why we did not observe significant associations between isolated living status and stress-related biomarkers.

Some studies have suggested that isolated living status is positively associated with physical disabilities. For instance, older people living in substandard neighborhoods have significantly higher incident mobility difficulties than those in less-deprived neighborhoods [47]. In our study, we did not find significant associations between different combinations of loneliness and isolated living status at baseline and mobility/IADL disability 6 years later after adjusting for baseline disabilities and covariates. The associations between isolated living status and physical disability in an aging population may be partly but not fully explained by correlated social and economic circumstances and social relationships [48].

### Strengths and limitations

The strengths of this analysis include the longitudinal design with a nationally representative sample cohort in Taiwan. The dataset provided multiple measures of health, demographic factors, and biological indicators for controlling for potential confounding variables. However, there are some limitations that should be noted. First, loneliness was assessed by using one question regarding the perception of loneliness in the past week. This measurement may be less reliable than a composite measurement of loneliness from multiple perspectives [49]. Secondly, compared to the complexity and inconsistency of social isolation measurements, the measurement of isolated living status in the present study may be a simpler indicator by which to explore the effects of objective isolation status on health [50]. However, the different effects of loneliness, isolated living status, and social isolation on health need more examination in future studies. Thirdly, some variables such as depressive symptoms and physical disability were addressed using self-reported rating scales, which may have led to response bias due to personality traits and anxiety [51]. Also, because the way people think about loneliness can be affected by age, sex, cross-cultural differences, and the cognitive/affective process of each individual, more research is needed to investigate whether our findings can be generalized to other populations [52]. Finally, this study only employed two waves of self-reported measurements and biomarkers, which may have fluctuated, so causality cannot be confirmed. Enrolling more waves for the purpose of measurement and checking diurnal changes in stress-related biomarkers may be a more convincing method by which to measure the effects of loneliness and isolated living status on health.

## Conclusion

This study simultaneously examines the effects of four combinations of loneliness and isolated living status on physical and mental health in longitudinal data, where each has been shown to have unique associations with levels of stress-related biomarkers (Cortisol, IL-6, and hsCRP), depressive symptoms, and physical disabilities (mobility and IADL). The findings suggested that those who felt lonely without being socially isolated had higher levels of cortisol and inflammatory markers than those who felt lonely and objectively isolated. However, only in the presence of both loneliness and isolated living status did depressive symptoms become more severe. In terms of physical disabilities, a positive association between loneliness, isolated living status, and physical disabilities did not exist after controlling for baseline and confounding variables. Based on our findings, we suggest that both loneliness and isolated living status be included in future studies to explore broader pathophysiological indicators for both physical and mental health.

## Data Availability

Data was publicly accessible with a request to the Ministry of Health and Welfare, Taiwan.

## Abbreviations

hsCRP: High-sensitivity C-reactive protein
IL-6: Interleukin-6
CRP: C-reactive protein
SEBAS: Social Environment and Biomarkers of Aging Study
TLSA: Taiwan Longitudinal Study on Aging
SPMSQ: Short Portable Mental State Questionnaire
PSS: Perceived Stress Scale
HPLC: High-performance liquid chromatography
EIA: Enzyme-linked immunoassays
ELISA: Enzyme-linked immunosorbent assays
CES-D: Center for Epidemiologic Studies Depression Scale
IADL: Instrumental Activities of Daily Living
SD: Standard deviation
CI: Confidence interval
